# RETRACTED: Paola et al. Environmental Impact of Pharmaceutical Pollutants: Synergistic Toxicity of Ivermectin and Cypermethrin. *Toxics* 2022, *10*, 388

**DOI:** 10.3390/toxics13020072

**Published:** 2025-01-22

**Authors:** Davide Di Paola, Carmelo Iaria, Fabio Marino, Enrico Gugliandolo, Cristian Piras, Rosalia Crupi, Salvatore Cuzzocrea, Nunziacarla Spanò, Domenico Britti, Alessio Filippo Peritore

**Affiliations:** 1 Department of Chemical, Biological, Pharmaceutical and Environmental Science, University of Messina, 98166 Messina, Italy; davide.dipaola@unime.it (D.D.P.); carmelo.iaria@unime.it (C.I.); fabio.marino@unime.it (F.M.); aperitore@unime.it (A.F.P.); 2 Department of Veterinary Science, University of Messina, 98166 Messina, Italy; egugliandolo@unime.it (E.G.); rcrupi@unime.it (R.C.); 3 Department of Health Sciences, “Magna Græcia University” of Catanzaro, Campus Universitario “Salvatore Venuta” Viale Europa, 88100 Catanzaro, Italy; cpiras@unicz.it (C.P.); britti@unicz.it (D.B.); 4 Department of Pharmacological and Physiological Science, Saint Louis University School of Medicine, St. Louis, MO 63104, USA; 5 Department of Biomedical and Dental Sciences and Morphofunctional Imaging, University of Messina, 98166 Messina, Italy

The journal retracts the article entitled “Environmental Impact of Pharmaceutical Pollutants: Synergistic Toxicity of Ivermectin and Cypermethrin” [1], which is cited above.

Following publication, concerns were brought to the attention of the Editorial Office regarding the duplication of images between this article [1] and other publications [2,3] produced by the same authorship group.

Adhering to our procedure on complaints, an investigation was conducted by the Editorial Office and the Editorial Board that confirmed the duplication of panels within Figures 2 and 3 published in [1] and panels in Figure 1 published in [2,3], showing different experimental conditions. Following discussions with the authors, the Editorial Board was unable to fully verify the validity of the duplicated images. Consequently, the Editorial Board has lost confidence in the reliability of the findings and decided to retract this publication [1], as per MDPI’s retraction policy (https://www.mdpi.com/ethics#_bookmark30). 

This retraction was approved by the Editor-in-Chief of the journal *Toxics*.

The authors did not agree to this retraction.
